# Transiliac-transsacral Screws: What is the Required Implant Length for Adequate Percutaneous Fixation of the Posterior Pelvic Ring?

**DOI:** 10.1055/s-0044-1800947

**Published:** 2025-04-28

**Authors:** Leonardo Comerlatto, Natália Henz Concatto, Marcus Vinícius Crestani, Tauã Brum Silva, Carlos Roberto Galia, Marco Aurélio Telöken

**Affiliations:** 1 Serviço de Ortopedia e Traumatologia, Hospital Moinhos de Vento, Porto Alegre, RS, Brasil; 2 Serviço de Radiologia, Hospital Moinhos de Vento, Porto Alegre, RS, Brasil

**Keywords:** bone screws, fracture fixation, internal, fractures, bone, pelvic bones

## Abstract

**Objective** Brazilian orthopedic surgeons experience the unavailability of long screws allowing percutaneous fixation of the posterior pelvic ring in transiliac-transsacral (TI-TS) configuration. The objective of the present study is to measure the lenght of the osseous fixation pathways available for TI-TS fixation in a population sample to infer the required implant length.

**Methods** We retrospectively assessed patients undergoing computed tomography (CT), initially identifying the existence of a potential osseous fixation pathway (POFP) in S1, S2 and S3. Each POFP was measured from the external cortex of the iliac bone to the external cortex of the contralateral iliac bone on axial CT images.

**Results** The analysis comprised a sample of 180 cases. A POFP was identified in S1 in 116 (64.4%) cases, in S2 in 178 (98.9%) cases, and in S3 in 16 (8.9%) cases. The median (interquartile range – IQR) POFP measurement in S1 was 153 (148–161) mm, ranging from 135 mm to 179 mm. In S2, the median (IQR) POFP measurement was 136 (131–144) mm, ranging from 114 to 160 mm. In S3, the median (IQR) POFP measurement was 120.5 (115–126) mm, ranging from 110 to 131 mm.

**Conclusions** We demonstrated that the maximum lengths of the osseous fixation pathways identified in our sample would require screws up to 180 mm in length, with a clear dissociation between the values measured and the longer screws currently commercialized in our setting.

## Introduction

From its first descriptions, 1 2 percutaneous fixation of traumatic posterior pelvic ring injuries using cannulated screws has been established as a safe, reproducible, effective, and versatile method, which can be used to treat a wide variety of fractures, dislocations, and fracture-dislocations affecting the posterior ilium, sacroiliac joint, and sacrum. 3 4 5 Current indications include more unstable injury patterns 4 6 and subjects with poor bone quality. 7 8 The method requires extensive knowledge of the anatomy, neurovascular structures at risk, and the osseous fixation pathways (OFP) available in each patient. 8 Sacral dysmorphism recognition is essential and guides the fixation strategy. 9

Depending on individual features and the purpose of implant use, implants can be positioned in the iliosacral (IS) or transiliac-transsacral (TI-TS) configuration. Screws in the traditional IS configuration are inserted from the iliac bone external cortex towards the sacral vertebral body. This configuration has proven insufficient, especially in situations of major instability, comminution, and osteoporosis. 10 11 The TI-TS configuration, i.e the insertion of a screw crossing the sacral segment and reaching the external cortex of the contralateral iliac bone, became possible only after the production of longer implants in the early 2000s. 12 Since then, a better understanding of OFPs and bone density-related particularities of the posterior ring have demonstrated clinical and biomechanical advantages of using screws in the TI-TS configuration. 13 14 15

Brazilian orthopedic surgeons experience the unavailability of cannulated screws with lengths that allow implant application in TI-TS configuration, preventing optimal OFP use and, in many cases, compromising the ability to offer sufficiently rigid and stable fixation.

The main objective of the present study was to perform the tomographic measurement of the length of OFPs used in the TI-TS fixation in a population sample.

## Materials and Methods

After obtaining Intitucional Review Board approval (CAAE: 78038724.5.0000.5330), we assessed retrospectively patients admitted in the emergency department between January 2nd and 10th, 2024, using the picture archiving and communication system (PACS) version 12 (Philips Medical Systems Nederland B.V., Best, The Netherlands) available at the institution.

Patients aged between 18 and 85 years who were evaluated in the hospital emergency department and underwent computed tomography (CT) of the abdomen/pelvis with serial axial images of up to 2 mm were included. Patients with traumatic injuries (acute or chronic) or malignancy-related anatomical deformities were excluded.

**Fig. 1:**
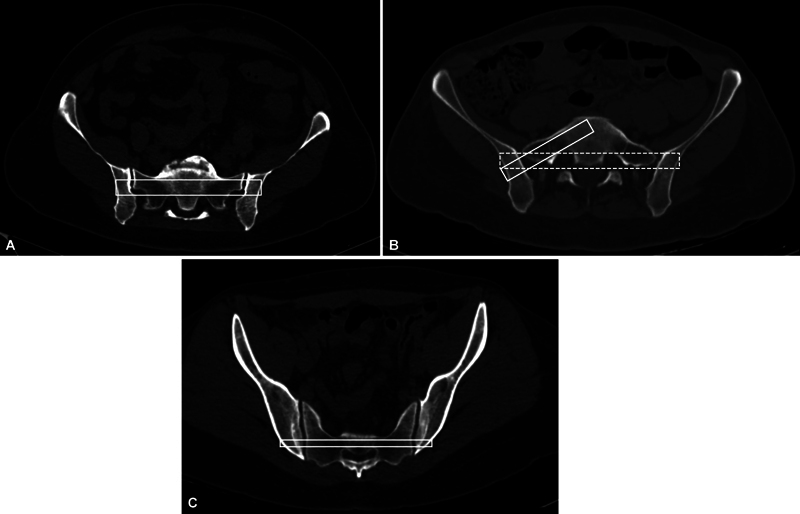
Definition of a transiliac-transsacral (TI-TS) potential osseous fixation pathway (POFP) in S1 and S3. (A) Presence of TI-TS POFP in S1 (absence of sacral dysmorphism). (B) The absence of TI-TS POFP in S1 (presence of sacral dysmorphism), with a narrow and oblique OFP at this level, only allows for implant application in the iliosacral (IS) configuration. (C) Patient with a TI-TS POFP in S3 presenting a bilateral complete articulation between the caudal portion of the iliac bone and the third sacral vertebra.

**Fig. 2:**
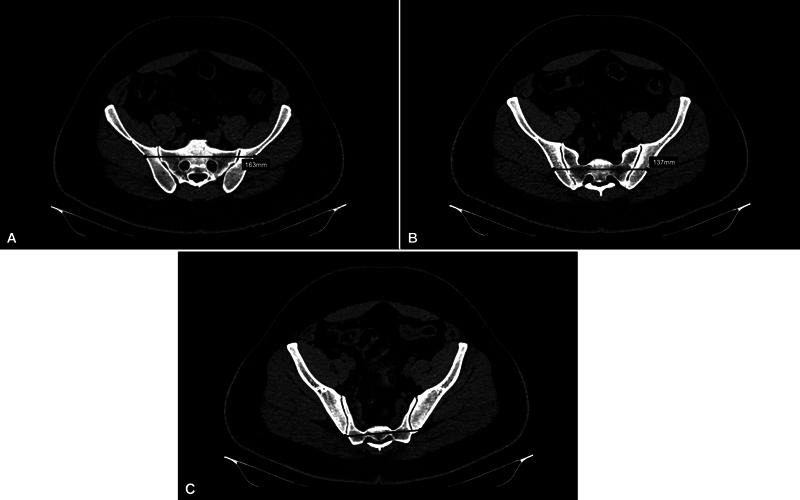
Technique for measuring the length of the potential osseous fixation pathway (POFP) in each patient. The patient has POFP in the first (A) and second (B) sacral segments, with lengths of 163 mm and 137 mm, respectively. There is no POFP in the third sacral segment (C).

The presence of a potential osseous fixation pathway (POFP) for a safe insertion of a TI-TS screw in the first (S1), second (S2), and third (S3) sacral segments was inittialy evaluated on axial CT images. The presence of POFP in S1 was defined by the existence of a wide bone corridor allowing for a safe insertion of a TI-TS screw according to previously described criteria; 16 these patients have been considered non-dysmorphic ( Figure 1A ). According to the same criteria, the presence of sacral dysmorphism is represented by the absence of a POFP in S1 that would allow the application of a TI-TS screw ( Figure 1B ). Only patients with a complete bilateral articulation between the caudal portion of the ilium and the third sacral vertebra were considered to have a POFP in S3 according to previous definition 17 ( Figure 1C ). POFPs identified in S1, S2, and S3 were measured (in millimeters *[mm]* ) from the external cortex of the posterior iliac bone to the external cortex of the contralateral posterior iliac to infer the minimum TI-TS configuration implant length ( Figure 2 ). The same evaluator (an orthopedic surgeon experienced in treating pelvic ring injuries) performed the measurements using the tool provided by the software.

All sample size calculations used the WinPEPI program (Programs for Epidemiologists for Windows) version 11.65 (Jerusalem, Israel) based on data from a pilot survey analyzing 20 patients and referring to subjects from both genders and the presence of PBCFs at each level. Considering the quantitative data for S1, the standard deviation for men (8.81) and women (7.10), an 80% analysis power, a 5% significance level, and a 10% potential loss margin, the minimum sample size required to detect a difference of five units between the groups was 63 participants from each gender, totaling 126 subjects.

Data processing, double entry into the database, review, and analysis used IBM SPSS Statistics for Windows (IBM Corp., version 21.0, Armonk, NY, USA) software. Descriptive analyses expressed quantitative data as mean ± standard deviation of the mean (± SD) or median and interquartile range (IQR) (25 ^th^ –75 ^th^ percentiles) per the Shapiro-Wilk distribution test. Qualitative variables were described as absolute frequencies (n) and relative frequencies (%). The Chi-squared (χ2) test with adjusted residual analysis assessed potential associations between qualitative variables. The Student's t-test for independent samples (t) compared means (± SD), and the Mann-Whitney (MW) test compared medians (IQR). For all analyses, the significance level was set at 5%.

## Results

The analysis comprised a sample of 180 patients admitted to the emergency department who underwent abdominal/pelvic CT between January 2nd and 10th, 2024. The sample had 115 women (63.9%) and 65 men (36.1%). The median (IQR) age was 43.5 years (34–62), ranging from 19 to 82. Of the total 180 cases included in the analysis, we identified a POFP in S1 in 116 (64.4%) cases, a POFP in S2 in 178 (98.9%) cases, and a POFP in S3 in 16 (8.9%) cases.

Table 1 shows the presence of POFP at each sacral level evaluated. We noted the absence of POFP at S1, i.e, the presence of sacral dysmorphism, in 64 (35.5%) of the 180 patients evaluated.

**Table 1:** Presence of a potential osseous fixation pathway at each sacral level evaluated

Sacral level	Total presence of POFP ( *N* = 180)
S1	116 (64.4)
S2	178 (98.9)
S3	16 (8.9)

Abbreviation: POFP, patients with a potential osseous fixation pathway.

Note: Data presented as absolute (n) and relative (%) frequencies.

Table 2 shows the length of each POFP in millimeters (mm) for each sacral level. The median [IQR] of the POFP at S1 was 153 (148–161) mm, ranging from 135 mm to 179 mm. At S2, the median (IQR) was 136 (131–144) mm, ranging from 114 to 160 mm. At S3, the median (IQR) POFP was 120.5 (115–126) mm, ranging from 110 to 131 mm.

**Table 2:** Lenght of the potencial osseous fixation pathways at each sacral level evaluated

Sacral level		POFP measurement (mm) ( *N* = 180)
S1 a	(minimum–maximum)	153 [148–160] (135–179)
S2 b	(minimum–maximum)	137.05 ± 8.98 (114–160)
S3 c	(minimum–maximum)	121.81 ± 6.92 (111–131)

Abbreviations: mm, millimeter; POFP, patients with a potential osseous fixation pathway.

Notes: Data presented as mean ± SD, median (IQR], minimum and maximum.

a *n* = 116 (64.4%).

b *n* = 178 (98.9%).

c *n* = 16 (8.9%).

Table 3 presents the differences between patients with and without sacral dysmorphism. Among those without sacral dysmorphism ( *N* = 116), the mean (± SD) of POFP length in S2 was 135.68 ± 8.54 mm, while, in those with sacral dysmorphism ( *N* = 64), the mean (± SD) POFP length in S2 was slightly higher, of 139.50 (± 9.30) mm (t, *p* = 0.006). There was a significant contrast between groups regarding the presence of POFP at S3. Among patients with sacral dysmorphism, 21.9% presented a POFP at S3, whereas only 1.7% of those without sacral dysmorphism did so (χ ^2^ , *p* ≤ 0.001).

**Table 3:** Differences between patients without and with sacral dysmorphism

	Without sacral dysmorphism (N = 116)	With sacral dysmorphism (N = 64)	* *p* -value
S2 measurement a	135.68 ± 8.54	139.50 ± 9.30	0.006
PCOF present in S3	2 (1.7)	14 (21.9)	≤ 0.001

Notes: Data presented as mean ± SD, absolute frequencies (n), and relative frequencies (%). Abbreviation: p – statistical significance index.

*Student's t-test for independent samples or Chi-squared test for adjusted residual analysis. Significance was set at 5% for all analyses.

a with sacral dysmorphism, *n* = 64 (100.0%); without sacral dysmorphism, *n* = 116 (98.3%).

Table 4 shows gender-related differences for each POFP at each sacral level evaluated. In S1, women exhibited a mean (± SD) length of 151.64 (± 9.19) mm, whereas men showed a mean (± SD) length of 157.14 (± 9.70) mm (t, *p* = 0.003). In S2, there was no significant difference between genders, with women presenting a mean (± SD) length of 137.21 (± 9.00) mm and men, 136.77 (± 9.01) mm (t, *p* = 0.752). In S3, although not statistically significant (MW, *p* = 0.055), there was a trend towards a difference, with women presenting a median (IQR) POFP length of 130 (121.5–130.5) mm and men 119 (116–123) mm.

**Table 4:** Measurement of the potential osseous fixation pathways at each sacral level according to gender

Sacral level	POFP measurement in females ( *N* = 115)	POFP measurement in males ( *N* = 65)	* *p* -value
S1	151.64 ± 9.19	157.14 ± 9.70	0.003
S2	137.21 ± 9.00	136.77 ± 9.01	0.752
S3	130 [121.5–130.5]	119 [116–123]	0.055

Abbreviations: *p* -value, statistical significance level; PBCF, patients with a potential osseous fixation pathway.

Notes: *Student's t-test for independent samples or Mann-Whitney test (MW).

Data presented as mean ± SD, or median (IQR).

Significance was set at 5% for all analyses.

Table 5 presents data on patients with POFP in S3. We identified 16 (8.9%) cases of POFP in S3, including 7 women (43.8%) and 9 men (56.3%). In addition, we noted morphology consistent with sacral dysmorphism in 14 cases (87.5%) and 2 cases (12.5%) had no sacral dysmorphism.

**Table 5:** Patients with a potential osseous fixation pathways in S3

Variable		POFP in S3 ( *n* = 16)
Gender	Female Male	7 (43.8) 9 (56.3)
Sacral dysmorphism	Yes No	14 (87.5) 2 (12.5)

Abbreviation: PBCF, patients with a potential bone corridor for fixation.

Note: Data presented as absolute (n) and relative (%) frequencies.

## Discussion

Screws in TI-TS configuration offer better fixation than in the IS configuration, and their insertion is preferred whenever possible according to the OFPs available in each patient. 13 14 15 16 Biomechanical studies comparing different posterior pelvic ring injuries fixation methods favor the use of TI-TS screws; 18 19 20 21 22 23 however, their use depends on the availability of implants of sufficient length. Long screws provide better load distribution, reducing stress at the tip of the implant and preventing secondary displacement. 12 TI-TS screws have higher pullout resistance, 15 allowing anchorage of up to six cortices; moreover, their higher lever arm increases shear resistance. 17 In addition to the absolute measurement of the implant length, fully threaded long screws may offer the possibility of anchoring the highest number of threads, contributing to their effectiveness in maintaining posterior ring stability. 12 Furthermore, the insertion of two TI-TS screws at the same sacral level has been described and is advantageous as long as it is carefully planned according to the published technique. 24

Recognition of sacral dysmorphism has fundamental practical implications for planning and executing percutaneous posterior ring fixation. Sacral dysmorphism is characterized by several radiographic peculiarities, such as non-circular sacral foramina and an acute alar slope. 25 However, in a more practical approach and with wider clinical applicability, a binary delineation is currently performed regarding the presence or absence of sacral dysmorphism. 13 16 The patient is defined as dysmorphic whenever the first sacral segment allows an intraosseous screw in the IS configuration but does not allow the safe intraosseous application of a TI-TS screw. In dysmorphic patients, the first sacral segment has a smaller and more obliquely oriented safe zone, allowing only the insertion of short screws in an oblique direction. In these subjects, S2 has a safety zone with a transverse orientation and a larger area 26 27 compared to the same segment in non-dysmorphic patients. The identification of sacral dysmorphism in 35.5% (64/180) of the patients analyzed is compatible with previously published data and, in this group, the impossibility of TI-TS fixation in S1 due to the lack of a safe OFP increases the significance of using S2 OFP.

Studies have shown that S3 may be a viable and safe option for TI-TS fixation in approximately 15% of patients, with dysmorphic patients being more likely to have a OFP at this level. 17 28 Our sample demonstrated the existence of POFP at S3 in only 16/180 (8.9%) cases and corroborated that most of these patients (87.5%) have a morphology consistent with sacral dysmorphism. When advanced intraoperative imaging techniques are available, 29 30 S3 offers an additional fixation site when a more stable construct is required. Although previously described, the insertion of TI-TS screws in S3 is compromised by the unavailability of a viable OFP in most of the population and the small number of studies defining its safety and indications. Furthermore, due to the high technical demand and precision required, as well as the structures at risk, percutaneous placement of screws in S3 presupposes the use of advanced intraoperative imaging techniques (such as three-dimensional fluoroscopy), which are rarely available in our setting.

Posterior pelvic ring percutaneous fixation requires a complete set of fully and partially threaded cannulated screws. 2 Although it is possible to apply implants with diameters ranging from 6.5 to 7.3 mm, 7.0-mm implants are the most widely available and studied. The production and commercialization of longer implants allowing TI-TS fixation occurred in major international centers only from the first decade of the 21 ^st^ century onwards. 12

Few scientific studies about this topic provide data specifically on screw lengths. Before the existence of longer screws and the popularization of the TI-TS fixation concept, Routt et al. 2 stated that screws up to 140 mm in length would be necessary to reach the contralateral sacral asa. In a study on percutaneous stabilization of “U”-shaped sacral fractures, Nork et al. 4 reported using screws of up to 150 mm. In a specific publication on TI-TS fixation, Gardner et al. 12 used implants of lengths ranging from 160 to 180 mm for S1 fixation and from 120 to 160 mm for S2 fixation.

**Fig. 3:**
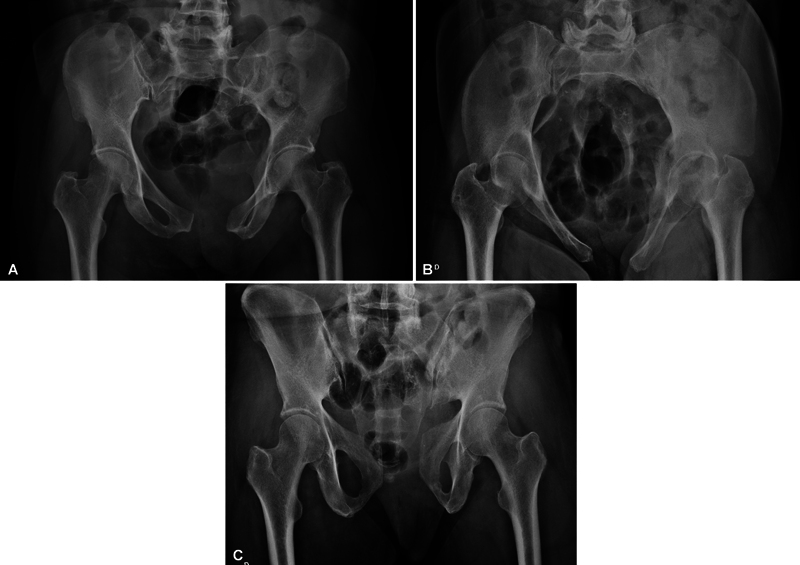
Case example (preoperative radiographs). Anteroposterior (AP) (A), inlet (B), and outlet (C) radiographs of a female patient presenting a mechanically unstable pelvic ring injury, including a complete and nondisplaced sacral fracture on the right side, incomplete injury of the left sacroiliac joint, and a pubic symphysis injury.

To the authors' knowledge, in the Brazilian federation unit where this study occurred, the longest 7.0-mm cannulated screws currently on the market are 150-mm long (partially threaded only). In the same federation unit, the most significant orthopedic trauma public institutions, which account for managing most patients with pelvic fractures, provided, at the time of this study, only partially threaded implants with a maximum length of 120 mm. We believe this reality extends to the rest of the country. Considering minimum and maximum lengths of S1 and S2 POFPs identified in our sample (from 135–179 mm for S1 and 114–160 mm for S2), we noticed the unavailability of implants allowing TI-TS fixation in most patients. In our practice, we have exceptionally identified patients with small dimensions allowing TI-TS fixation in S2. In the case presented Figures 3 4 5 6 7 (images from author's archive), preoperative planning indicated no sacral dysmorphism, with a wide OFP in S1, measuring 141 mm, and representing a length higher than the longer implant offered by the institution at that time. On the other hand, S2 OFP measured 118 mm, which allowed for planning and safe insertion of a 120 mm TI-TS screw without a washer.

**Fig. 4:**
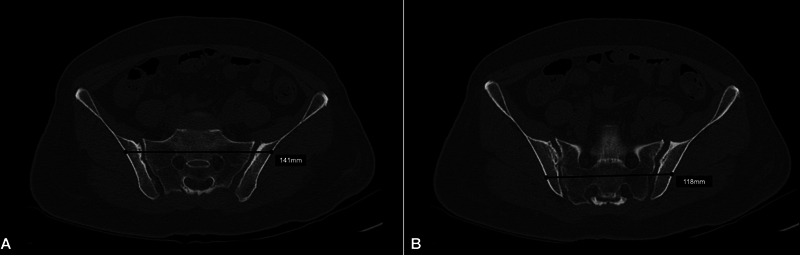
Case example (preoperative planning). Axial computed tomography scans demonstrating the measurement of the length of the bone corridors for fixation in the first (A) and second (B) sacral segments during preoperative planning.

**Fig. 5:**
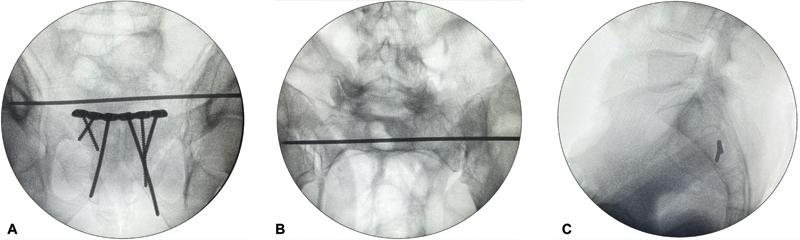
Case example (intraoperative images). Fluoroscopic (A) outlet, (B) inlet, and (C) lateral views of the sacrum acquired intraoperatively.

**Fig. 6:**
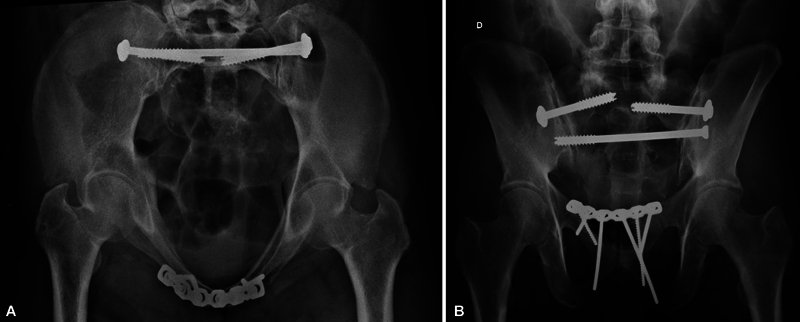
Case example (postoperative radiographs). Postoperative images in inlet (A) and outlet (B) views demonstrating the iliosacral fixation in S1 bilaterally and transiliac-transsacral fixation in S2.

**Fig. 7:**
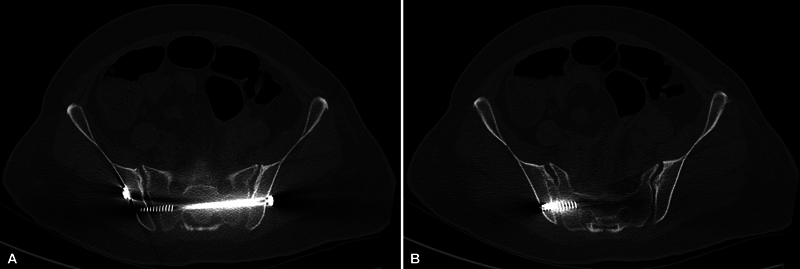
Case example (postoperative computed tomography [CT] scans). CT images demonstrating the safe and intraosseous positioning of the previously planned implant in S2.

Limitations of this study include the fact that planning TI-TS screw requires additional sagittal plane evaluation. 16 The proposed measurement (between the external cortices of the posterior iliac as a way of determining the implant length) occurred in axial images alone and, therefore, we preferred the term “potential” OFP. It is worth mentioning that the measurement technique used in this study does not consider that, conceptually, an implant should exceed one or two threads the opposite cortex. In addition, there is a preference for using washers, which would increase the real implant length in a clinical situation by around 2 or 3 mm.

## Conclusion

Percutaneous posterior pelvic ring fixation is a traditional, well-established method widely validated by clinical and biomechanical studies. From a historical perspective, it is clear that the production of longer implants for screw application in TI-TS configuration allowed the method to establish itself as the main method of treatment for traumatic injuries affecting the posterior iliac, sacroiliac joint, and sacrum.

In our sample, we demonstrated that the OFP lenghts are dissociated from the longer screws currently commercialized in our country, and an adequate percutaneous posterior pelvic ring fixation requires a complete set of cannulated screws with lengths of up to 180 mm.

## References

[1] Matta J M Saucedo T Internal fixation of pelvic ring fractures Clin Orthop Relat Res 1989 242 83 97 2706863

[2] Routt M L Meier M C Kregor P J Mayo K A Percutaneous iliosacral screws with the patient supine technique Oper Tech Orthop 1993 3 01 35 45

[3] Routt M L Jr Kregor P J Simonian P T Mayo K A Early results of percutaneous iliosacral screws placed with the patient in the supine position J Orthop Trauma 1995 9 03 207 214 7623172 10.1097/00005131-199506000-00005

[4] Nork S E Jones C B Harding S P Mirza S K Routt M L Jr Percutaneous stabilization of U-shaped sacral fractures using iliosacral screws: technique and early results J Orthop Trauma 2001 15 04 238 246 11371788 10.1097/00005131-200105000-00002

[5] Calafi L A Routt M L Jr Posterior iliac crescent fracture-dislocation: what morphological variations are amenable to iliosacral screw fixation? Injury 2013 44 02 194 198 23182751 10.1016/j.injury.2012.10.028

[6] Saiz A M Jr Kellam P J Amin A Percutaneous sacral screw fixation alone sufficient for mildly displaced U-type sacral fractures with preserved osseous fixation pathways Eur J Orthop Surg Traumatol 2023 10.1007/s00590-023-03661-4 PMC11490423 37874399

[7] Cintean R Fritzsche C Zderic I Gueorguiev-Rüegg B Gebhard F Schütze K Sacroiliac versus transiliac-transsacral screw osteosynthesis in osteoporotic pelvic fractures: a biomechanical comparison Eur J Trauma Emerg Surg 2023 49 06 2553 2560 37535095 10.1007/s00068-023-02341-6 PMC10728224

[8] Bishop J A Routt M L Jr Osseous fixation pathways in pelvic and acetabular fracture surgery: osteology, radiology, and clinical applications J Trauma Acute Care Surg 2012 72 06 1502 1509 22695413 10.1097/TA.0b013e318246efe5

[9] Kaiser S P Gardner M J Liu J Routt M L Jr Morshed S Anatomic Determinants of Sacral Dysmorphism and Implications for Safe Iliosacral Screw Placement J Bone Joint Surg Am 2014 96 14 e120 25031382 10.2106/JBJS.M.00895

[10] Tabaie S A Bledsoe J G Moed B R Biomechanical comparison of standard iliosacral screw fixation to transsacral locked screw fixation in a type C zone II pelvic fracture model J Orthop Trauma 2013 27 09 521 526 23114418 10.1097/BOT.0b013e3182781102

[11] Salazar D Lannon S Pasternak O Investigation of bone quality of the first and second sacral segments amongst trauma patients: concerns about iliosacral screw fixation J Orthop Traumatol 2015 16 04 301 308 26018428 10.1007/s10195-015-0354-y PMC4633427

[12] Gardner M J Routt M L Jr Transiliac-transsacral screws for posterior pelvic stabilization J Orthop Trauma 2011 25 06 378 384 21577075 10.1097/BOT.0b013e3181e47fad

[13] Eastman J G Shelton T J Routt M LC Jr Adams M R Posterior pelvic ring bone density with implications for percutaneous screw fixation Eur J Orthop Surg Traumatol 2021 31 02 383 389 32902718 10.1007/s00590-020-02782-4

[14] Beaulé P E Antoniades J Matta J M Trans-sacral fixation for failed posterior fixation of the pelvic ring Arch Orthop Trauma Surg 2006 126 01 49 52 16311761 10.1007/s00402-005-0069-2

[15] Chang G Fram B Sobol K Krieg J C Two Transiliac-Transsacral Screws in a Single Sacral Level: Surgical Technique and Patient Outcomes Tech Orthop 2021 36 01 50

[16] Lucas J F Routt M L Jr Eastman J G A Useful Preoperative Planning Technique for Transiliac-Transsacral Screws J Orthop Trauma 2017 31 01 e25 e31 27661733 10.1097/BOT.0000000000000708

[17] Eastman J G Adams M R Frisoli K Chip Routt M L Jr Is S3 a Viable Osseous Fixation Pathway? J Orthop Trauma 2018 32 02 93 99 29065034 10.1097/BOT.0000000000001036

[18] Zhao Y Zhang S Sun T Mechanical comparison between lengthened and short sacroiliac screws in sacral fracture fixation: a finite element analysis Orthop Traumatol Surg Res 2013 99 05 601 606 23850128 10.1016/j.otsr.2013.03.023

[19] Min K S Zamorano D P Wahba G M Garcia I Bhatia N Lee T Q Comparison of two-transsacral-screw fixation versus triangular osteosynthesis for transforaminal sacral fractures Orthopedics 2014 37 09 e754 e760 25350616 10.3928/01477447-20140825-50

[20] Chen P H Chen C Y Lin K C Hsu C J Quantification of the Safe Zone of the First to Third Sacral Segments for Transiliac-Transsacral Screw Fixation in Normal and Dysmorphic Sacra Orthopedics 2024 47 01 e13 e18 37276441 10.3928/01477447-20230531-06

[21] Jazini E Klocke N Tannous O Does Lumbopelvic Fixation Add Stability? A Cadaveric Biomechanical Analysis of an Unstable Pelvic Fracture Model J Orthop Trauma 2017 31 01 37 46 27997465 10.1097/BOT.0000000000000703

[22] Gonçalves R M Freitas A Aragão V AD Comparison of sacroiliac screw techniques for unstable sacroiliac joint disruptions: a finite element model analysis Injury 2023 54 06 110783 38143127 10.1016/j.injury.2023.05.014

[23] Collinge C A Crist B D Combined Percutaneous Iliosacral Screw Fixation With Sacroplasty Using Resorbable Calcium Phosphate Cement for Osteoporotic Pelvic Fractures Requiring Surgery J Orthop Trauma 2016 30 06 e217 e222 26741641 10.1097/BOT.0000000000000520

[24] Schultz B J Mayer R M Phelps K D Assessment of sacral osseous fixation pathways for same-level dual transiliac-transsacral screw insertion Arch Orthop Trauma Surg 2023 143 10 6049 6056 37103608 10.1007/s00402-023-04892-0

[25] Miller A N Routt M L Jr Variations in sacral morphology and implications for iliosacral screw fixation J Am Acad Orthop Surg 2012 20 01 8 16 22207514 10.5435/JAAOS-20-01-008

[26] Gardner M J Morshed S Nork S E Ricci W M Chip Routt M L Jr Quantification of the upper and second sacral segment safe zones in normal and dysmorphic sacra J Orthop Trauma 2010 24 10 622 629 20871250 10.1097/BOT.0b013e3181cf0404

[27] Conflitti J M Graves M L Chip Routt M L Jr Radiographic quantification and analysis of dysmorphic upper sacral osseous anatomy and associated iliosacral screw insertions J Orthop Trauma 2010 24 10 630 636 20871251 10.1097/BOT.0b013e3181dc50cd

[28] Hwang J S Reilly M C Shaath M K Safe Zone Quantification of the Third Sacral Segment in Normal and Dysmorphic Sacra J Orthop Trauma 2018 32 04 178 182 29401088 10.1097/BOT.0000000000001100

[29] Shaw J Gary J Ambrose C Routt M C Multidimensional pelvic fluoroscopy: A new and novel technique for assessing safety and accuracy of percutaneous iliosacral screw fixation J Orthop Trauma 2020 34 11 572 577 33065656 10.1097/BOT.0000000000001796

[30] Warner S J Haase D R Chip Routt M L Eastman J G Achor T S Use of 3D Fluoroscopy to Assist in the Reduction and Fixation of Pelvic and Acetabular Fractures: A Safety and Quality Case Series J Orthop Trauma 2023 37(11S):S1 S6 10.1097/BOT.0000000000002686 37828694

