# Supplementary material for: Transiliac-transsacral Screws: What is the Required Implant Length for Adequate Percutaneous Fixation of the Posterior Pelvic Ring?
Source: Rev Bras Ortop (Sao Paulo). 2025 Apr 28;60(1):s00441800947. doi: 10.1055/s-0044-1800947 (PMC12037221; doi:10.1055/s-0044-1800947)
Supplement: Related texts

# Research article: Parafusos transilíaco-transacrais: Qual o comprimento necessário dos implantes para uma adequada fixação percutânea do anel pélvico posterior?

**Keywords:** fixação interna de fraturas, fraturas ósseas, ossos pélvicos, parafusos ósseos

## Abstract

**Objetivo** Cirurgiões ortopédicos brasileiros vivenciam a indisponibilidade de parafusos longos que permitam a fixação percutânea do anel pélvico posterior na configuração transilíaco-transacral (TI-TS). O objetivo do presente estudo é mensurar o comprimento dos corredores ósseos de fixação disponíveis para fixação TI-TS em uma amostra populacional para inferir o comprimento necessário do implante.

**Métodos** Foram avaliados retrospectivamente pacientes que realizaram tomografia computadorizada (TC), identificando inicialmente a existência de um potencial corredor ósseo de fixação (PCOF) em S1, S2 e S3. Cada PCOF foi medido a partir da cortical externa de um ilíaco até a cortical externa do ilíaco contralateral em imagens axiais de TC.

**Resultado** A análise compreendeu uma amostra de 180 casos. PCOF em S1 foi identificado em 116 (64,4%) casos, PCOF em S2 foi identificado em 178 (98,9%) casos e PCOF em S3 foi identificado em 16 (8,9%) casos. A mediana (intervalo interquartílico - IIQ) da aferição do PCOF de S1 foi de 153 (148–161) mm, variando de 135 a 179 mm. Em S2, a mediana (IIQ) foi de 136 (131–144) mm, com uma variação de 114 a 160 mm. Em S3, a mediana (IIQ) da medição do PCOF foi de 120,5 (115–126) mm, com uma variação de 110 a 131 mm.

**Conclusões** Demonstramos que os comprimentos máximos dos corredores ósseos de fixação identificados exigiriam parafusos de até 180 mm de comprimento, com uma clara dissociação entre os valores medidos e os parafusos mais longos atualmente comercializados em nosso meio.

## Introdução

Desde suas primeiras descrições, 1 2 a fixação percutânea das lesões traumáticas do anel pélvico posterior utilizando parafusos canulados se estabeleceu como um método seguro, reprodutível, efetivo e versátil, possuindo aplicação em uma ampla variedade de fraturas, luxações e fraturas-luxações que acometem o ilíaco posterior, articulação sacroilíaca e sacro. 3 4 5 As indicações atuais incluem padrões mais instáveis de lesão, 4 6 bem como pacientes com má qualidade óssea. 7 8 O emprego do método exige amplo conhecimento da anatomia, das estruturas neurovasculares em risco e dos corredores ósseos de fixação (COFs) disponíveis em cada paciente. 8 O reconhecimento do dismorfismo sacral é fundamental e direciona a estratégia de fixação. 9

De acordo com as características individuais e o objetivo da sua utilização, os implantes podem ser posicionados na configuração iliossacral (IS) ou transilíaco-transacral (TI-TS). Parafusos na tradicional configureção IS são inseridos do córtex externo do osso ilíaco em direção ao corpo vertebral sacral. Essa configuração demonstrou-se insuficiente, especialmente em situações de maior instabilidade, cominuição e osteoporose. 10 11 A configuração TI-TS, ou seja, a inserção de um parafuso cruzando o segmento sacral e alcançando o córtex externo do osso ilíaco contralateral, tornou-se possível apenas após a produção de implantes mais longos no início dos anos 2000. 12 Desde então, o melhor entendimento dos COFs e das particularidades relacionadas à densidade óssea do anel posterior vêm demonstrando vantagens clínicas e biomecânicas da utilização de parafusos na configuração TI-TS. 13 14 15

Cirurgiões ortopédicos brasileiros vivenciam a indisponibilidade de parafusos canulados de comprimentos que permitam a aplicação rotineira de implantes na configuração TI-TS, impedindo a utilização ideal dos COFs e, em muitos casos, comprometendo a capacidade de oferecer uma fixação suficientemente rígida e estável.

O objetivo principal deste estudo é realizar, em uma amostra populacional, a aferição tomográfica do comprimento dos COFs utilizados na fixação TI-TS.

## Materiais e Métodos

Após aprovação do comitê de ética institucional (CAAE: 78038724.5.0000.5330), foi realizada avaliação retrospectiva de pacientes atendidos no setor de emergência no período entre 2 e 10 de janeiro de 2024 através do *picture archiving and communication system* (PACS) version 12 (Philips Medical Systems Nederland B.V, Best, Holanda) disponível na instituição.

Foram incluídos pacientes com idades entre 18 e 85 anos que foram avaliados no pronto-socorro do hospital e realizaram tomografia computadorizada (TC) de abdome/pelve com imagens axiais seriadas de até 2 mm. Pacientes com lesões traumáticas (agudas ou crônicas) ou deformidades anatômicas relacionadas a malignidades foram excluídos.

A presença de um potencial corredor ósseo de fixação (PCOF) para a inserção segura de um parafuso TI-TS no primeiro (S1), segundo (S2) e terceiro (S3) segmentos sacrais foi inicialmente avaliada em imagens axiais de TC. A presença de PCOF em S1 foi definida pela existência de um amplo corredor ósseo que permite a inserção segura de um parafuso TI-TS, de acordo com os critérios previamente descritos; 16 esses pacientes foram considerados não dismórficos ( Figura 1A ). Conforme o mesmo critério, a presença de dismorfismo sacral é representada pela ausência de um PCOF em S1 que permitiria a aplicação de um parafuso TI-TS ( Figura 1B ). Apenas pacientes com uma completa articulação entre a porção caudal do ilíaco e a terceira vértebra sacral bilateralmente foram considerados como possuindo um PCOF em S3, conforme definição previamente publicada 17 ( Figura 1C ). Os PCOFs identificados em S1, S2 e S3 foram medidos (em milímetros [mm]) do córtex externo do osso ilíaco posterior até o córtex externo do osso ilíaco posterior contralateral para inferir o comprimento mínimo do implante na configuração TI-TS ( Figura 2 ). O mesmo avaliador (um cirurgião ortopédico experiente no tratamento de lesões do anel pélvico) realizou as medições utilizando a ferramenta fornecida pelo software.

**Fig. 1:** Definição da presença de potencial corredor ósseo de fixação (PCOF) transilíaco-transacral (TI-TS) em S1 e S3. (A) Presença de PCOF TI-TS em S1 (ausência de dismorfismo sacral). (B) Ausência de PCOF TI-TS em S1 (presença de dismorfismo sacral), existindo neste nível corredor ósseo estreito e obliquado que permitiria apenas a aplicação de implantes na configuração iliosacral (IS). (C) Paciente com presença de PCOF TI-TS em S3 caracterizado pela identificação de uma completa articulação entre a porção caudal do ilíaco e a terceira vértebra sacral bilateralmente.

**Fig. 2:** Técnica utilizada para a aferição do comprimento dos potenciais corredores ósseos de fixação (PCOFs) identificados em cada paciente. O paciente possui PCOF no primeiro (A) e segundo (B) segmentos sacrais, com comprimentos de 163 mm e 137 mm, respectivamente. Não há PCOF no terceiro segmento sacral (C).

Para todos os cálculos do tamanho da amostra, utilizou-se o programa WinPEPI (Programas para Epidemiologistas para Windows) versão 11.65 (Jerusalem, Israel), baseado nos dados de um levantamento piloto que analisou 20 pacientes, referentes a homens e mulheres e presença de PCOFs em cada nível. Considerando os dados quantitativos para S1: desvio-padrão de homens (8.81) e mulheres (7.10), um poder de análise de 80%, um nível de significância de 5% e uma margem de 10% para possíveis perdas, o tamanho amostral mínimo necessário para detectar uma diferença de 5 unidades entre os grupos é de 63 participantes em cada sexo, totalizando 126 sujeitos.

O software IBM SPSS Statistics for Windows (IBM Corp., versão 21.0, Armonk, NY, EUA) foi utilizado para o processamento, dupla entrada no banco, revisão e análise dos dados. Foram conduzidas análises descritivas, com os dados quantitativos expressos como média ± desvio padrão da média (± DP) ou mediana e intervalo interquartílico (IIQ) (percentis 25–75), conforme o teste de distribuição Shapiro-Wilk. As variáveis qualitativas foram descritas em termos de frequências absolutas (n) e frequências relativas (%). Possíveis associações entre variáveis qualitativas foram analisadas utilizando o teste do Qui-quadrado (χ ^2^ ) com análise residual ajustada. As comparações entre médias (± DP) foram conduzidas utilizando o teste t de Student para amostras independentes (t), e entre medianas (IIQ) utilizando o teste de Mann-Whitney (MW). Para todas as análises, o nível de significância foi fixado em 5%.

## Resultados

A análise compreendeu uma amostra de 180 pacientes admitidos no pronto-socorro que realizaram tomografia computadorizada abdominal/pélvica entre 2 e 10 de janeiro de 2024. A amostra consistiu em 115 mulheres (63,9%) e 65 homens (36,1%). A mediana (IIQ) de idade foi de 43,5 (34–62) anos, variando de 19 a 82.

Do total de 180 casos incluídos na análise, um PCOF em S1 foi identificado em 116 (64,4%) casos, um PCOF em S2 foi identificado em 178 (98,9%) casos e um PCOF em S3 foi identificado em 16 (8,9%) casos. A presença de PCOF em cada nível sacral avaliado está ilustrada na Tabela 1 . A inexistência de PCOF em S1, ou seja, a presença de dismorfismo sacral, foi identificada em 64 (35,5%) dos 180 pacientes avaliados.

**Tabela 1:** Presença de potencial corredor ósseo de fixação em cada nível sacral avaliado

Nível sacral	Total de presença de PCOF ( *N* = 180)
S1	116 (64.4)
S2	178 (98.9)
S3	16 (8.9)

Abreviatura: PCOF, pacientes com um potencial corredor ósseo de fixação.

Nota: Dados apresentados como frequências absolutas (n) e relativas (%).

A medida do comprimento de cada PCOF em milímetros (mm), em cada nível sacral avaliado, está fornecida na Tabela 2 . A mediana (IIQ) da aferição do PCOF em S1 foi de 153 (148–161) mm, variando de 135 mm a 179 mm. Em S2, a mediana (IIQ) foi de 136 (131–144) mm, com uma variação de 114 a 160 mm. Em S3, a mediana (IIQ) da medição de PCOF foi de 120,5 (115–126) mm, com uma variação de 110 a 131 mm.

**Tabela 2:** Comprimento do potencial corredor ósseo de fixação em cada nível sacral avaliado.

Nível sacral		Medida do PCOF (mm) ( *N* = 180)
S1 a	(mínimo–máximo)	153 [148–160] (135–179)
S2 b	(mínimo–máximo)	137.05 ± 8.98 (114–160)
S3 c	(mínimo–máximo)	121.81 ± 6.92 (111–131)

Abreviaturas: mm, milímetro; PCOF, pacientes com um caminho potencial de fixação óssea.

Nota: Dados apresentados como média ± DP, mediana [IIQ], mínimo e máximo.

a *n* = 116 (64.4%).

b *n* = 178 (98.9%).

c *n* = 16 (8.9%).

A Tabela 3 apresenta as diferenças entre pacientes com e sem dismorfismo sacral. Entre aqueles sem dismorfismo sacral ( *N* = 116), a medição média (± DP) do PCOF de S2 foi de 135,68 ± 8,54 mm, enquanto naqueles com dismorfismo sacral ( *N* = 64), a medição média (± DP) do PCOF de S2 foi ligeiramente maior, 139,50 (± 9,30) mm (t, *p* = 0,006). Houve contraste significativo entre os grupos quanto à presença de PCOF em S3. Dentre os pacientes com dismorfismo sacral, 21,9% apresentaram um PCOF em S3, enquanto apenas 1,7% daqueles sem dismorfismo sacral o fizeram (χ ^2^ , *p* ≤ 0,001).

**Tabela 3:** Diferenças entre pacientes sem e com dismorfismo sacral

	Sem dismorfismo sacral (N = 116)	Com dismorfismo sacral (N = 64)	*valor de *p*
Medida de S2 a	135.68 ± 8.54	139.50 ± 9.30	0.006
Presença de PCOF em S3	2 (1.7)	14 (21.9)	≤ 0.001

Abreviatura: *p* , índice de significância estatística.

Notes: Dados apresentados como média ± DP, frequências absolutas (n), e frequências relativas (%). *Teste t de Student para amostras independentes ou teste do Qui-quadrado para análise ajustada residual. Significância definida em 5% para todas as análises.

a n = com dismorfismo sacral *n* = 64 (100.0%), sem dismorfismo sacral *n* = 116 (98.3%).

As diferenças relacionadas ao gênero em cada PCOF em cada nível sacral avaliado estão exibidas na Tabela 4 . Em S1, as mulheres exibiram uma média (± DP) de 151,64 (± 9,19) mm, enquanto os homens mostraram uma média (± DP) de 157,14 (± 9,70) mm (t, *p* = 0,003). Em S2 não houve diferença significativa entre os gêneros, com as mulheres apresentando uma média (± DP) de 137,21 (± 9,00) mm e os homens 136,77 (± 9,01) mm (t, *p* = 0,752). Em S3, embora não estatisticamente significativo (MW, *p* = 0,055), houve uma tendência para uma diferença, com as mulheres apresentando uma mediana [IIQ] de PCOF de 130 (121,5–130,5) mm e os homens 119 (116–123) mm.

**Tabela 4:** Medida dos potenciais corredores ósseos de fixação em cada nível sacral de acordo com o gênero

Nível sacral	Medida do PCOF nas mulheres (N = 115)	Medida do PCOF nos homens ( *N* = 65)	*valor de *p*
S1	151.64 ± 9.19	157.14 ± 9.70	0.003
S2	137.21 ± 9.00	136.77 ± 9.01	0.752
S3	130 [121.5–130.5]	119 [116–123]	0.055

Abreviaturas: *p* , índice de significância estatística; PCOF, pacientes com um caminho potencial de fixação óssea.

Notas: *Teste t de Student para amostras independentes ou teste de Mann-Whitney (MW).

Dados apresentados como média ± DP, ou mediana [IIQ].

Significância definida em 5% para todas as análises.

A Tabela 5 apresenta dados sobre pacientes com presença de PCOF em S3. Foram identificados 16 (8,9%) casos com presença de PCOF em S3, sendo observadas 7 mulheres (43,8%) e 9 homens (56,3%). Morfologia compatível com dismorfismo sacral foi identificada em 14 casos (87,5%), enquanto 2 casos (12,5%) não apresentaram dismorfismo sacral.

**Tabela 5:** Pacientes com potencial corredor ósseo de fixação em S3

Variável		Presença de PCOF em S3 ( *n* = 16)
Gênero	Mulheres Homens	7 (43.8) 9 (56.3)
Dismorfismo sacral	Sim Não	14 (87.5) 2 (12.5)

Abreviatura: PCOF, potencial corredor ósseo de fixação.

Nota: Dados apresentados como frequências absolutas (n) e relativas (%).

## Discussão

Parafusos na configuração TI-TS oferecem melhor fixação do que na configuração IS, e sua inserção é preferida sempre que possível, de acordo com os PCOFs disponíveis em cada paciente. 13 14 15 16 Estudos biomecânicos que comparam diferentes métodos de fixação de lesões do anel pélvico posterior favorecem o uso de parafusos TI-TS 18 19 20 21 22 23 no entanto, seu uso depende da disponibilidade de implantes de comprimento suficiente. Parafusos longos possuem maior capacidade de distribuição de carga, reduzindo o estresse na ponta do implante e prevenindo deslocamento secundário. 12 Os parafusos TI-TS têm maior resistência ao arrancamento, 15 permitindo a ancoragem de até seis corticais; além disso, seu maior braço de alavanca aumenta a resistência ao cisalhamento. 17 Além da medida absoluta do comprimento do implante, parafusos longos totalmente rosqueados oferecem a possibilidade de ancoragem do maior número de roscas, contribuindo para a sua efetividade na manutenção da estabilidade do anel posterior. 12 Ademais, a inserção de dois parafusos TI-TS em um mesmo nível sacral é descrita e demonstrou-se vantajosa desde que criteriosamente planejada conforme técnica publicada. 24

O reconhecimento do dismorfismo sacral possui implicações práticas fundamentais para o planejamento e execução da fixação percutânea do anel posterior. O dismorfismo sacral é caracterizado por várias peculiaridades radiográficas, como forames sacrais não circulares e uma inclinação aguda das asas. 25 Porém, de uma forma mais prática e com maior aplicabilidade clínica, realiza-se atualmente o delineamento binário quanto à presença ou não de dismorfismo sacral. 13 16 O paciente é definido como dismórfico sempre que o primeiro segmento sacral permite um parafuso intraósseo na configuração IS, mas não possibilita a aplicação intraóssea segura de um parafuso TI-TS. No paciente dismórfico, o primeiro segmento sacral possui uma zona segura menor e mais obliquamente orientada, sendo possível apenas a inserção de parafusos curtos e com direcionamento oblíquo. Já o segundo segmento sacral nos pacientes dismórficos apresenta uma zona de segurança com orientação transversa e de maior área 26 27 quando comparado ao mesmo segmento em pacientes não-dismórficos. A identificação de dismorfismo sacral em 35,5% (64/180) dos pacientes analisados é compatível com dados publicados anteriormente e, neste grupo, a impossibilidade de fixação TI-TS em S1 devido à falta de um COF seguro aumenta a importância do uso do COF em S2.

Estudos demonstraram que S3 pode ser uma opção viável e segura para fixação TI-TS em aproximadamente 15% dos pacientes, sendo que os dismórficos apresentam maior probabilidade de apresentar um COF neste nível. 17 28 Nossa amostra demonstrou a existência de PCOF em S3 em apenas 16/180 (8,9%) dos casos e corroborou que a maioria desses pacientes (87,5%) apresentam morfologia compatível com dismorfismo sacral. Quando técnicas avançadas de imagem intraoperatória estão disponíveis, 29 30 S3 oferece um ponto adicional de fixação quando uma construção mais estável é necessária. Embora tenha sido descrita anteriormente, a inserção de parafusos TI-TS em S3 é comprometida pela indisponibilidade de um COF viável na maioria da população e pelo pequeno número de estudos que definem sua segurança e indicações. Além disso, devido à alta demanda técnica e à precisão necessária, bem como às estruturas em risco, a colocação percutânea de parafusos em S3 pressupõe o uso de técnicas avançadas de imagem intraoperatória (como fluoroscopia tridimensional), as quais são raramente disponíveis em nosso ambiente.

A fixação percutânea do anel pélvico posterior requer um conjunto completo de parafusos canulados, totalmente e parcialmente roscados. 2 Implantes de diâmetros entre 6,5 e 7,3 mm podem ser utilizados, porém os de 7,0 mm são os mais disponíveis e estudados. A produção e comercialização de implantes longos que possibilitassem a fixação TI-TS ocorreu nos grandes centros internacionais somente a partir da primeira década dos anos 2000.

Poucos estudos científicos sobre este tema fornecem dados especificamente sobre os comprimentos dos parafusos. Em uma época anterior à existência de parafusos longos e à popularização do conceito de fixação TI-TS, Routt et al. 2 afirmaram que parafusos de até 140 mm de comprimento seriam necessários para alcançar a asa sacral contralateral. Em estudo sobre a estabilização percutânea de fraturas em “U” do sacro, Nork et al. 4 relataram o uso de parafusos de até 150 mm. Gardner et al., 12 em uma publicação específica sobre a fixação TI-TS, apresentaram dados nos quais implantes de comprimentos entre 160 e 180 mm foram utilizados na fixação de S1, enquanto implantes entre 120 e 160 mm foram utilizados na fixação de S2.

Ao conhecimento dos autores, na unidade da federação brasileira onde o presente estudo foi desenvolvido, os parafusos canulados de 7,0 mm mais longos atualmente comercializados possuem 150 mm de comprimento (parcialmente roscados apenas). Na mesma unidade da federação, as instituições públicas de trauma ortopédico mais significativas, responsáveis pelo atendimento da maioria dos pacientes com fraturas da pelve, forneciam, na época deste estudo, apenas implantes com rosca parcial e comprimento máximo de 120 mm, realidade que acreditamos estender-se ao restante do território nacional. Considerando as medidas mínimas e máximas do comprimento dos PCOFs de S1 e S2 identificadas na amostra de pacientes apresentada (135–179 mm para S1 e 114–160mm para S2), percebemos a indisponibilidade de implantes que possibilitem a fixação TI-TS na maior parte dos pacientes. Em nossa prática, excepcionalmente, identificamos pacientes de dimensões pequenas nos quais há possibilidade de fixação TI-TS no S2. No caso apresentado nas Figuras 3 4 5 6 7 (imagens do arquivo do autor principal), o planejamento pré-operatório indicou ausência de dismorfismo sacral, com um amplo COF em S1, medindo 141 mm, e representando um comprimento superior ao do implante mais longo oferecido pela instituição na época. Por outro lado, o COF em S2 media 118 mm, o que permitiu o planejamento e a inserção segura de um parafuso TI-TS de 120 mm sem arruela.

**Fig. 3:** Caso exemplo (radiografias pré-operatórias). Radiografias anteroposterior (AP) (A), *inlet* (B) e *outlet* (C) de uma paciente do sexo feminino apresentando uma lesão mecanicamente instável do anel pélvico, incluindo uma fratura sacral completa e não deslocada no lado direito, lesão incompleta da articulação sacroilíaca esquerda e uma lesão na sínfise púbica.

**Fig. 4:** Caso exemplo (planejamento pré-operatório). Cortes axiais da tomografia computadorizada demonstrando a aferição do comprimento dos corredores ósseos de fixação no primeiro (A) e segundo (B) segmentos sacrais durante o planejamento pré-operatório.

**Fig. 5:** Caso exemplo (imagens intraoperatórias). Incidências fluoroscópicas de (A) *outlet* , (B) *inlet* e (C) perfil do sacro adquiridas no intraoperatório.

**Fig. 6:** Caso exemplo (radiografias pós-operatórias). Imagens pós-operatórias nas incidências *inlet* (A) e *outlet* (B) demonstrando fixação iliossacral em S1 bilateralmente e transilíaco-transacral em S2.

**Fig. 7:** Caso exemplo (TC pós-operatória). Imagens tomográficas demonstrando posicionamento seguro e intraósseo do implante previamente planejado em S2.

As limitações deste estudo incluem o fato de que o planejamento do parafuso TI-TS requer uma avaliação adicional no plano sagital. 16 A medição proposta (entre as corticais externas dos ilíacos posteriores como forma de determinar o comprimento do implante) ocorreu apenas em imagens axiais e, portanto, preferimos o termo "potencial" COF. Vale a pena mencionar que a técnica de medição utilizada neste estudo não considera que, conceitualmente, um implante deve ultrapassar uma ou duas roscas o córtex oposto. Além disso, há uma preferência pelo uso de arruelas, o que aumentaria o comprimento real do implante em uma situação clínica em cerca de 2 ou 3 mm.

## Conclusões

A fixação percutânea do anel pélvico posterior é um método tradicional, bem estabelecido e amplamente validado por estudos clínicos e biomecânicos. Do ponto de vista histórico, é claro que a produção de implantes mais longos para a aplicação de parafusos na configuração TI-TS permitiu que o método se estabelecesse como o principal método de tratamento para lesões traumáticas que afetam o ilíaco posterior, a articulação sacroilíaca e o sacro.

Na amostra de pacientes reportada, demonstramos que os comprimentos dos COFs estão dissociados dos parafusos mais longos comercializados em nosso meio na atualidade e, para uma adequada fixação percutânea do anel pélvico posterior, é necessário um conjunto completo de parafusos canulados com comprimentos de até 180 mm.
